# The interpretation of proverbs by elderly with high, medium and low educational level: Abstract reasoning as an aspect of executive functions

**DOI:** 10.1590/S1980-57642011DN05010006

**Published:** 2011

**Authors:** Thalita Bianchi de Oliveira Wachholz, Mônica Sanches Yassuda

**Affiliations:** 1 Graduate in Gerontology from the Escola de Artes, Ciências e Humanidades da Universidade de São Paulo, São Paulo SP, Brazil.; 2 Associate Professor, PhD, of the Gerontology Course of the Escola de Artes, Ciências e Humanidades da Universidade de São Paulo, São Paulo SP, Brazil.

**Keywords:** aging, executive functions, abstract reasoning, test of proverbs

## Abstract

It is now known that cognitive functions tend to decline with age. Executive functions (EF) are among the first abilities to decline with aging. A subcomponent of the EF is abstract reasoning. The Test of Proverbs is an instrument that can be used to evaluate the capacity of abstract reasoning.

**Objective:**

To examine the association of performance in interpretation of proverbs, with education and with episodic memory and EF tasks.

**Methods:**

A total of 67 individuals aged between 60 and 75 years were evaluated, and divided into three categories of education: 1-4 years, 5-8 years, and 9 or more years of schooling. The instruments used were a sociodemographic questionnaire (gender, age, marital status, education, income, previous occupation, current occupation and health perception), the Mini Mental State Examination, Brief Cognitive Screening Battery; Geriatric Depression Scale; Forward and Backward Digit Span (WAIS-III), and the Test of Proverbs.

**Results:**

A high impact of education was seen on the interpretation of proverbs, with lower performance among the elderly with less education. A significant association between performance on the Test of Proverbs and scores on the MMSE, GDS, and verbal fluency tests was found. There was a modest association with incidental memory.

**Conclusions:**

The capacity to interpret proverbs is strongly associated with education and with performance on other EF tasks.

## Abstract (pt)

Sabe-se atualmente que funções cognitivas tendem a declinar com a idade, e as funções executivas (FE) incluem algumas das primeiras habilidades a declinar no envelhecimento. Um subcomponente das FE é a capacidade do raciocínio abstrato. Um instrumento utilizado para avaliar o desempenho na capacidade de abstração é o Teste de Provérbios.

**Objetivo:**

Examinar a associação entre o desempenho na interpretação de provérbios com a escolaridade e com tarefas de memória episódica e de FE.

**Métodos:**

Foram avaliados 67 idosos, com idade entre 60 e 75 anos, divididos em três faixas de escolaridade: entre 1 a 4 anos, 5 a 8 anos, e com 9 ou mais de escolaridade. Os instrumentos utilizados foram: questionário sociodemográfico (sexo, idade, estado civil, escolaridade, renda, ocupação anterior, ocupação atual e percepção da saúde); Mini Exame do Estado Mental (MEEM); Bateria Breve de Rastreio Cognitivo (BBRC); Escala de Depressão Geriátrica (GDS); Dígitos Diretos e Inversos (WAIS-III); Teste de interpretação de provérbios.

**Resultados:**

Foi observado efeito significativo da escolaridade sobre a interpretação dos provérbios, com menor desempenho entre os idosos com menor escolaridade. Encontrou-se também associação significativa entre o desempenho no teste dos provérbios e o MEEM, a GDS, e a fluência verbal. Houve associação de menor magnitude com a memória incidental.

**Conclusões:**

A capacidade de interpretar provérbios está fortemente associada à escolaridade e ao desempenho em outras tarefas de FEs.

The aging process is a universal phenomenon which affects all living creatures and is associated with alterations in physiological functions. Cerebral functions, are also affected by aging.^1^

The effects of aging on cognition display inter- and intra-subject variation.^2^ Some cognitive functions decline with age while others are preserved or improve.^3^ Functions that are maintained during aging include motor skills, autobiographic memory, semantic knowledge (vocabulary, language comprehension and reading), and the ability to recall information through pre-activation. Functions which tend to decline during the aging process include the ability to learn unfamiliar content, complex language expression, and abstract reasoning.^4^

In a review of the frontal lobe theory in cognitive aging, West^5^ highlighted that the cognitive processes subserved by the frontal lobes, and more specifically the pre-frontal cortex, are among the first to undergo changes with increasing age. Such cognitive processes, commonly called executive functions (EF), are among the first skills to decline in aging.^6^

The concept of executive functions (EF) is broad and differs widely. According to Oliveira-Souza,^7^ EF denotes a group of mental operations which organize and guide the various cognitive domains so they can function in a biologically adaptive manner. Kristensen^8^ defines EF as cognitive processes of control and integration aimed at performing task-driven behavior. EFs rely on the performance of several sub-components such as attention, programming and planning of sequences, inhibition of processes and concurrent information, and monitoring. These processes are closely related to the activation of the frontal lobes such that any lesions in this area of the brain, or aging-related alterations to the frontal lobes, can impair performance on certain skills.^9^

One aspect which warrants special attention in the study of EF is abstract reasoning, which can be regarded as a sub-component of executive functions. Abstract reasoning is characterized by the ability to resolve problems involving abstract symbols,^10^ such as the task of interpreting proverbs. Interpreting proverbs has a long tradition in assessing abstract thought, particularly in schizophrenia. Although the use of proverb interpretation as a diagnostic tool has been called into question over the years, comprehending non-literal (figurative) language plays an important role in social interactions.^11^

The aim of the present study was to explore the relationship between the interpretation of proverbs and schooling, and also to investigate the relationship of the interpretation of proverbs with other cognitive functions, in elders grouped by schooling into three educational categories.

## Methods

### Participants

A total of 67 elders were recruited, aged between 60 and 75 years, and divided according to three schooling levels: Group 1, with 1 to 4 years of schooling (19 participants), Group 2, 5 to 8 years (22 participants), and Group 3, 9 years or more (26 participants). The individuals were contacted and invited to take part in the study while frequenting the Open University for the Third Age of the University of São Paulo’s School of Arts, Sciences and Humanities (EACH USP) to do physical, educational or social activities. Individual interviews were held in a separate room after signing of a Free and Informed Consent Term, each lasting approximately 30 minutes. None of the elderly participants in the study sample had possible dementia or depression on the screening tests applied (see below).

### Materials

The initial assessment involved the application of a socio-demographic questionnaire used to collect information related to gender, age, marital status, schooling, income, health perception and satisfaction with health.

Subsequently, the Mini Mental State Exam^12^ was applied to characterize the sample and screen for possible dementias. The Brief Cognitive Screening Battery^13^ was used to assess naming, episodic memory and executive functions. This battery entails presenting of a board with 10 black and white drawings of common figures. The individual is asked to name the 10 items (Naming). Next, the sheet is withdrawn and the participant is asked to recall which figures were on the sheet within a 1-minute period (Incidental Memory). The sheet is then presented again with the instruction that the items must be memorized by the individual for 30 seconds. The sheet is again withdrawn, and the subject requested to recall the figures (Immediate memory). This procedure is repeated once more (Learning).

Two other tests are then applied: Verbal Fluency (animals) in one minute, which assesses language, semantic memory and executive functions, and the Clock Drawing Test (CDT), evaluating executive functions and visuo-constructive abilities. After around five minutes have elapsed, the time needed for the last two tests, the examiner asks the individuals to recall the figures presented earlier, allowing 1 minute to do so (Delayed Memory). In the last stage, a sheet containing 20 figures is shown. Ten of the figures are those shown earlier while the other 10 are distractor figures. The task is to recognize the previously presented figures (Recognition).

The Geriatric Depression Scale^14^ (GDS) was applied to quantify depression symptoms. In order to assess attention and working memory, the Forward and Backward Digit Span test from the WAIS-III (Wechsler Adult Intelligence Scale)^15^ battery was employed. The interviewee was asked to repeat several sequences of digits, first in forward order and then other sequences in reverse order. The sequences of digits to be repeated were presented in increasing order of difficulty.

Lastly, the proverb interpretation test by Silva^16^ was applied. This test requires the subject to interpret 20 popular proverbs. After the proverbs are read, the interviewee chooses one of three possible answers: one of the options is irrelevant, one represents a literal interpretation and the other corresponds to an abstract interpretation (correct). The score was calculated based on the number of abstract answers chosen, with each correct answer scoring one point.

### Statistical analyses

Descriptive analyses were used to characterize the sample. Given the majority of the variables did not have a normal distribution, non-parametric tests were adopted. Fisher’s exact test was used to compare categorical variables among schooling categories. The Kruskal-Wallis test was used to compare the groups for numeric variables. Spearman’s correlation coefficient was used to analyze the relationship among numeric variables. The level of significance adopted for the statistical tests was 5% (p<0.05).

Univariate and multivariate linear regression analyses with Stepwise variable selection criteria were used to examine the relationship between the dependent variable (proverbs) and independent variables (years of schooling, health assessment, satisfaction with health, MMSE, Naming, Incidental Memory, Immediate Memory, Learning, Delayed Memory, Recognition, Verbal Fluency, CDT, GDS, Forward and Backward Digit Span). Stepwise Forward modeling was elected. Variables yielding a value of p<0.20on the simple linear regression analysis were included in the multiple models. The data were input to the Excel program, 2007 version. The SPSS v.17.0 software package and the Statistica v.7.0 program were used for the statistical analysis.

## Results

Table 1 shows the sociodemographic profile (age, schooling, income and proportion of women) of the sample, the results of the health assessment and satisfaction, as well as scores on the Geriatric Depression Scale by schooling level. The mean age of the three groups (G1=1-4 years of schooling, G2=5-8 years, and G3=9 or more years) was 66.31 years (SD=5.18), and no significant difference among the groups was found for age. Mean years of schooling was 8.34 years (SD=4.17), and G3 had the greatest level of schooling. Regarding income, individuals with greater schooling reported higher incomes. Concerning health assessment, G1 had the lowest level compared to G2 and G3. For satisfaction with health, Groups G1 and G3 reported greater satisfaction than did G2. No statistical difference in GDS score was found among the groups. The sample comprised predominantly women.

**Table 1:** Characterization of sample by schooling level, mean and standard deviation (in brackets).

	1-4 years (n=19)	5-8 years (n=22)	9 years or + (n=26)	p-value
Age	66.58 (6.23)	66.05 (4.14)	66.35 (5.32)	0.984^a^
Schooling	3.47 (0.86)	7.18 (1.18)	12.88 (1.90)	<0.001^a^
Health assessment	3.42 (0.76)	4.27 (0.55)	4.12 (0.65)	<0.001^a^
Satisfaction with health	3.47 (0.841)	4.23 (0.528)	3.92 (0.628)	0.004ª
GDS	2.47 (1.71)	1.82 (1.94)	1.65 (1.49)	0.189^a^
Income (%)				<0.001^b^
Less than 1 MW	10.5	4.5	7.7	
From 1.5 to 3 MWs	68.4	40.9	23.1	
From 3.5 to 5 MWs	21.1	36.4	23.1	
More than 5 MWs	0	18.2	46.1	
No. of women (%)	15 (78.9)	17 (77.3)	18 (69.2)	0.315^b^

a Kruskal-Wallis: for significant values, test was followed by multiple comparisons z' values. MW: minimum wages; G1: 1-4 years of schooling; G2: 5-8 years, G3: 9 or +. Schooling: G1<G2 (p=0.002); G1<G3 (p<0.001); G2<G3 (p<0.001). Health assessment: G1<G2 (p=0.004); G1<G3 (p<0.022); G2=G3 (p=1.000). Satisfaction with health: G1<G2 (p=0.017); G1=G3 (p=0.313); G2=G3 (p=0.575).

b Fisher's Exact Test.

Table 2 shows the results of the cognitive variables by schooling level. A statistically significant difference was detected among the groups for: MMSE, Incidental Memory, Verbal Fluency, Forward and Backward Digit Span, and Proverbs. Lower performance was observed in the group with least schooling (G1). For Incidental Memory and Proverbs, the group with lowest schooling had worse performance compared to the other two groups (G2 and G3). Regarding Verbal Fluency and Backward Digit Span, the least-schooled group (G1) had a poorer performance than the most-schooled group (G3)

**Table 2:** Mean and standard deviation (in brackets) for cognitive variables of the three schooling groups.

	1-4 years (n=19)	5-8 years (n=22)	9 years or + (n=26)	p-value^a^
MMSE	26.00 (2.35)	27.59 (1.70)	28.27 (1.53)	0.003^c^
Naming	10.00 (0.00)	10.00 (0.00)	10.00 (0.00)	1.000
Inc. memory	5.26 (1.24)	6.50 (1.73)	6.35 (1.01)	0.009^b^
Immed. memory	8.21 (1.27)	8.64 (1.56)	8.46 (0.98)	0.296
Learning	9.32 (0.67)	9.50 (0.74)	9.27 (0.82)	0.499
Delayed memory	8.95 (1.02)	8.86 (1.32)	9.00 (1.16)	0.933
CDT	4.26 (0.87)	4.50 (0.85)	4.73 (0.45)	0.144
Verbal fluency	14.68 (3.56)	17.45 (3.91)	19.38 (4.63)	0.001^c^
Digit Span				
Forward	8.84 (3.27)	9.00 (2.07)	9.27(2.70)	0.646
Backward	4.00 (2.16)	4.77 (1.95)	5.81 (2.11)	0.010^c^
Proverbs	13.21 (3.85)	16.91 (2.56)	18.73 (1.82)	<0.001^b^

a Kruskal-Wallis: for significant values, test was followed by multiple comparisons z' values.

b G1<G2, G1<G3, G2=G3;

c G1=G2, G1<G3, G2=G3. G1=1-4 years of schooling, G2=5-8 years, G3=9 or +.

Table 3 shows Spearman’s correlations between proverbs and the independent variables. Results revealed a significant correlation between proverbs and the variables: years of schooling, MMSE score, Incidental Memory, Verbal Fluency, and Backward Digit Span.

**Table 3:** Spearman's Correlations between Proverbs Test and independent variables.

Proverbs	rho*	p-value
Years of schooling	0.63	<0.001
Health assessment	0.18	0.140
Satisfaction with health	0.04	0.766
MMSE	0.58	<0.001
Naming	0.00	1.000
Incidental memory	0.25	0.041
Immediate memory	0.10	0.416
Learning memory	0.06	0.650
Delayed memory	0.19	0.117
Recognition	0.12	0.353
Verbal fluency	0.43	<0.001
CDT	0.21	0.091
GDS	0.06	0.632
Forward Digit Span	0.14	0.268
Backward Digit Span	0.31	0.011

* Correlation Coefficient: Spearman's rho.

Univariate and Multivariate linear regression analyses were carried out (Tables 4 and 5, respectively) to assess the relationship among the independent variables and the proverbs test. On univariate regression, schooling level, MMSE score, Incidental Memory, Verbal Fluency and GDS score, were found to be factors associated to performance on the Proverbs Test.

**Table 4:** Univariate regression analysis for Proverb Test scores including sociodemographic and cognitive variables.

Dependent variable	Predictive factors	Beta	SE Beta	p-value
Proverbs	Schooling (years)	0.335	0.138	0.019
	Gender (ref. women)	-0.076	0.098	0.442
	Age	-0.028	0.103	0.790
	Income	0.068	0.123	0.583
	Health assessment	0.139	0.125	0.271
	Satisfaction with health	-0.103	0.113	0.367
	MMSE	0.280	0.116	0.020
	Incidental memory	0.218	0.108	0.050
	Immediate memory	-0.183	0.113	0.112
	Learning memory	-0.022	0.112	0.842
	Delayed memory	0.187	0.109	0.093
	Recognition	0.118	0.107	0.276
	Verbal fluency	0.228	0.112	0.047
	CDT	0.027	0.106	0.802
	GDS	0.265	0.104	0.014
	Forward Digit Span	0.027	0.123	0.825
	Backward Digit Span	-0.041	0.133	0.759

Note: R^2^ (adjusted)=0.49; p-value (model)<0.001.

**Table 5:** Multivariate linear regression analysis by Stepwise Forward model (p<0.20), for Proverb Test scores including sociodemographic and cognitive variables.

Dependent variable	Predictive factors	Beta	SE Beta	p-value
Proverbs	Schooling (years)	0.437	0.103	<0.001
	MMSE	0.229	0.101	0.027
	Delayed memory	0.127	0.091	0.167
	GDS	0.239	0.092	0.012
	Verbal fluency	0.198	0.096	0.043
	Incidental memory	0.157	0.093	0.098

Note: R^2^ (adjusted)=0.52; p-value (model)<0.001.

Multivariate regression analysis with the Stepwise Forward model was employed to ascertain which of these predictive factors, taken together, were most strongly correlated with proverb scores, and to verify whether any previously significant variables were no longer associated to performance on the proverbs test in the presence of the other variables. The results in Table 5 indicate that MMSE score, GDS score, Verbal Fluency and particularly, schooling level, were the factors most strongly associated with proverb test performance.

## Discussion

The aim of the present article was to determine the relationship between proverb interpretation and schooling. Another aim was to investigate the relationship between proverb interpretation and other cognitive functions. The results showed that comprehension and interpretation of proverbs was related to schooling of each individual, since significant differences among schooling levels in proverb interpretation were evident, and schooling was associated to this performance on the regression analyses. Proverb interpretation was also shown to be associated with other tests assessing EF and general mental status.

The finding of an association between schooling and proverb interpretation is in line with results of a study by Uekermann et al.,^6^ whose regression analysis confirmed the influence of schooling on proverb comprehension. Nippold et al.^17^ also detected the influence of schooling on proverb comprehension, reporting that throughout lifespan a slight decline in this skill occurs from the age of 60 years, reaching statistical significance after 70 years of age. The results of the present study are congruent with previously cited data. However, our study did not present data on age since the age variability in the study sample was relatively limited (from 60 to 75 years). Age was capped at 75 years because the aim of this study was not to investigate the impact of age on proverb interpretation. Future investigations should examine the impact of aging on this cognitive skill through cross-sectional and longitudinal studies.

Banhato and Nascimento^18^ described the effect of schooling on cognitive tasks involving EF in older adults, such as Verbal Fluency, Matrix Reasoning, Symbol Digit, Block Design and Digits. Notably, the effect of schooling in the present study was also significant for MMSE score, Verbal Fluency test and Backward Digit Span which, similarly to the Proverb test, also assess aspects of EF. These results suggest that EFs in general are modulated by schooling, perhaps to a more significant degree than episodic memory, as previous reports have indicated.^19^

Argimon and Stein^20^ presented similar data, describing that older adults who had up to three years of education achieved a significantly poorer result on the MMSE scale and Digit Span tests than did elders with four or more years of schooling. In the above-mentioned study, no statistical difference for Verbal Fluency was found among different schooling groups, conflicting with earlier data from the literature.^13,21^ This result might be explained by the fact that the sample did not vary greatly in schooling level.

With regard to correlations between proverb interpretation and the other variables, the study found a significant relationship with schooling, MMSE, GDS, Verbal Fluency and the Inverse Digit span test. On memory tests, a significant but weaker correlation was detected only for Incidental Memory.^19^ Remarkably, incidental memory can be considered a manifestation of implicit memory, and distinct from episodic memory.

The results of this study, and those of other investigations in the literature,^6,22,23^ suggest that EFs are more closely related to working memory (short term) than to episodic memory (long term). By the same logic, there appears to be a weaker relationship between proverb interpretation and episodic memory yet a stronger relationship with functions associated to the EF concept. An explanation for this result may lie in the fact that the brain regions recruited in episodic memory tasks differ to those recruited for EF tasks. As outlined earlier, EFs are related to the frontal lobes, more specifically to the pre-frontal cortex^5,7,9,24,25^ whereas episodic memory processes are more related to the medial temporal lobes such as the hippocampus, entorhinal cortex and amygdala.^26^ The capacity for abstract reasoning, assessed by the Proverbs Test, as a well as by Verbal Fluency, are aspects of EF supported by frontal lobe functioning, and a correlation between these skills is therefore to be expected.

Nevertheless, some studies have shown that frontal lobes are involved in many aspects of episodic memory, and there is evidence frontal lobes and medial temporal lobes work in conjunction to consolidate the storage of long-term memory. Patients with damage to the pre-frontal regions for instance, do not develop drastic episodic memory loss, but do tend to have serious difficulties in recalling the time sequence of events.^26^ Episodic memory performance is dependent on the integrity of the pre-frontal regions of the brain.^22,23^

Research evidence suggests that adequate understanding and use of figurative language is affected in cases of cognitive deficit and that the Proverb test can help identify dementias and other diseases.^6^ In an effort to investigate the pattern of performance among healthy elderly on abstraction and reasoning tests, Matsdorff et al.^27^ applied proverb interpretation tests and noted that elderly often made literal interpretations. Determining proverb comprehension in the elderly is important to identify cognitive decline, and consequent difficulties in social relationships among this group, given that, according to the literature on the theory of the mind,^6,7,28^ social and occupational competence rely on abstract reasoning.

This study had several limitations, including small sample size and absence of an assessment of the participants’ previous level of familiarity with proverbs. Indeed, the more highly-schooled individuals may have been more familiar with proverbs, thereby influencing the results. Notwithstanding, it is clear that the interpretation of proverbs is a valid approach for assessing EF, and that this tasks is strongly affected by schooling. In addition, the results point to the fact that abstract reasoning has scant connection with figure memorization processes. Further studies are warranted in the area, preferably using the Proverbs Test by Silva,^16^ to corroborate the findings reported. Silva’s Proverb Test should also be tested as a screening instrument for cognitive loss, provided that schooling effects are taken into account. Proverbs interpretation activities can also be part of cognitive rehabilitation protocols.
